# Production, Optimization, Characterization and Drought Stress Resistance by β-Glucan-Rich Heteropolysaccharide From an Endophytic Fungi *Colletotrichum alatae* LCS1 Isolated From Clubmoss (*Lycopodium clavatum*)

**DOI:** 10.3389/ffunb.2021.796010

**Published:** 2022-01-04

**Authors:** Hiran Kanti Santra, Debdulal Banerjee

**Affiliations:** Microbiology and Microbial Biotechnology Laboratory, Department of Botany and Forestry, Vidyasagar University, Midnapore, India

**Keywords:** β-glucan heteropolysaccharide, drought resistance, endophyte, *Colletotrichum alatae*, sustainable yield

## Abstract

Endophytic entities are ubiquitous in nature with all-square bioactivity ranging from therapeutic effects toward animals to growth promoting attributes and stress tolerance activities in case of green plants. In the present study, the club moss *Lycopodium clavatum* for the first time has been subjected for the isolation of endophytic fungi. An exopolysaccharide (EPS) extracted from *Colletotrichum alatae* LCS1, an endophytic fungi isolated from *L. clavatum* Linn., was characterized as a β-glucan heteropolymer (composed of mannose, rhamnose, arabinose, glucose, galactose, and fucose) which plays a pivotal role in obliterating the drought stress in rice seedlings (*Oryza sativa*) when applied at an amount of 20, 50, and 100 ppm. The fresh weight contents of rice tissue (39%), total chlorophyll (33%), proline (41%), soluble sugar content (26%) along with antioxidant enzymes such as catalase, peroxidase, and super-oxide dismutase increased (in comparison to control of non-EPS treated seedlings) while malondialdehyde content had reduced markedly after 30 days of regular treatment. The drought resistance of rice seedling was observed at peak when applied at 50 ppm dosage. Vital parameters for EPS production like fermentation duration (5 days), medium pH (6), nutrient (carbon (glucose-7 g%/l), nitrogen (yeast extract-0.4 g%/l), and mineral (NaCl-0.10 g%/l) sources, oxygen requirements (O_2_ vector or liquid alkane-n-hexane, n-heptane, n-hexadecane), and headspace volume (250 ml Erlenmeyer flask- 50 ml medium, 200 ml-headspace volume) were optimized to obtain an enhanced EPS yield of 17.38 g/L−59% higher than the preoptimized one. The present study, for the first time, reported the β-glucan rich heteropolysaccharide from Colletotrichum origin which is unique in structure and potent in its function of drought stress tolerance and could enhance the sustainable yield of rice cultivation in areas facing severe drought stress.

## Introduction

Endophytes are the hidden microbial entities that reside within the different parts of host tissues as a symbiotic partner of the plant taxa. Secondary metabolites from endophytes of fungal and bacterial origin possess wide spectrum of biological activity. They can act as antimicrobial agents and antioxidative therapeutics that can promote plant growth and development under abiotic stress conditions like high salinity, drought, and heavy metal toxicity (Santra and Banerjee, 2020a,b). Endophytic microbial flora mediate plant growth promotions, ameliorate microbial parasitic infections, alleviate stresses, and enhance plant vigor by a variety of means like ACC-1-aminocyclopropane 1 carboxylate deaminase activity (lowers the levels of stress inducer ethylene), siderophore production, phytohormones synthesis, increased rate of photosynthesis, altered root morphology, and emission of Volatile organic compounds (VOCs) (Santra and Banerjee, 2021).

In recent past, the overexploitation of natural resources, global warming, and water scarcity is posing a serious threat to the existence of the valuable plant species on earth and consequently affects overall agricultural production. To cope with this adverse situation, sustainable agricultural approaches are found to be the best figured solution. The deep ecological concepts are the crying need of the 21st century in order to maintain a sustainable self-renewable eco-system. So, it is of utmost need to have plant varieties that can have drought-salt-cold resistance and, at the same time, maintains the quality and quantity of yielding crops, but it is a time taking process and cannot be found promptly. But here, microbial symbionts can be put forward to establish a potent plant-microbe relationship where microbial fermentation products can be widely utilized in order to alleviate stress conditions in a habitat-specific manner. Here, polymers of biological origin can play a crucial role as they can not only modify the soil texture and form viscous film to retain soil moisture but also can provide a positive change on the physical, bio-chemical, and genetic aspects of the plant treated with them.

Polysaccharides are a particular class of carbohydrate polymers that are obtained from microbial communities, plants, and from animal tissues. Microbial populations being easy to handle is the primary source for extracellular polymeric substances. In this study, fungal exopolysaccharide has been extracted and characterized. Exopolysaccharides are the extracellular polymeric substances that are produced during logarithmic phase of the microorganism's growth cycle and performs unique biological functions. They are valuable therapeutic agents in the pharmaceutical industry and are a highly demanded component in food and cosmetics. A variety of monosaccharide composition and glycosidic linkages have made it a valuable tool and improves its physiochemical properties. EPSs are popular in pharmaceuticals industry as food stabilizers, thickeners, solidifiers, viscosity enhancers, and ion chelators. Anti-tumor, anti-cancer, anti-proliferative, anti-inflammatory properties, and probiotics effect of microbial EPSs have been reported widely by various authors (Chen et al., 2018; Liu et al., 2018).

Exopolysaccharides (EPS) are the most suitable targets for endophyte biologists due to their easy accessibility and economic feasibility in terms of extraction, optimization, and purification.

Rice seedlings treated with *Pantoeaalhagi* (endophyte of Leguminous plant *Alhagisparsifolia* Shap.) exopolysaccharide exhibits drought stress alleviation and salt stress tolerance. The biochemical characters, the antioxidant defense enzymes (catalase, per-oxidase, and super-oxide dismutase), malondialdehyde contents, proline contents, and soluble sugar parameters were modified to tolerate salt and drought stress. The physical conditions (fresh weight and relative water contents) of the seedlings were improved under exopolysaccharide treatment in comparison to the control group (Chen et al., 2017). Other examples include the utilization of endophytic bacterial exopolysaccharides (*Pseudomonas aeruginosa* PF23, and *Kosakonia* sp.) in drought stress and salt stress alleviation in case of sunflowers and wheat plants where they act as osmo-protective and antioxidative activity enhancers and water retention agent (Tewari and Arora, 2014; Gao et al., 2019).

In this study, an endophyte, *Colletotrichum alatae* LCS1 MH102383.1, isolated from a pteridophytic ancient herb *Lycopodium clavatum*, collected from Tapobon forest region (a virgin forest patch of Eastern India) was evaluated for its exopolysaccharide production and the produced β-glucan-rich EPS was found to be a mixture of six monosaccharides, namely, glucose, galactose, mannose, fucose, rhamnose, and arabinose (glucose being the prime component), with unique *in vivo* drought stress tolerance potency. The exopolysaccharides, when applied as foliar spray in varying concentrations of 20, 50, and 100 ppm on PEG mediated drought-stress-induced rice seedlings (*Oryza sativa* ssp. *indica* MTU7093 Swarna), exhibits drought stress amelioration property. The treated plants are found to have improved physical (relative water contents and fresh weight of tissues) and biochemical characters (chlorophyll contents, proline, and soluble sugar amounts) in comparison to the untreated control seedlings. The treatment groups showed enhancement of antioxidant defense enzymes, namely, catalase, peroxidase, super oxide dismutase and a reduced level of malonaldehyde contents. The essential fermentation parameters, namely, fermentation time, fermentation volume, dissolved oxygen amount, additional carbon source, nitrogen source, appropriate mineral salt concentration, and medium pH, was optimized. O_2_ vectors (n-hexane, n-heptane, and n-hexadecane) were also added to the fermentation medium for better transport of oxygen to speed up the exopolysaccharide production. It can be safely said that this type of study opens up new horizon in the field of sustainable agricultural practices using endophyte biology as a potent tool.

It is a matter of great importance that the present study bears novelty regarding the study area (virgin spot of Tapobon forest patch) regarding selection of rare ethnic lycopod genus, optimization, characterization, and drought stress alleviation of exopolysaccharide from *C*. *alatae* LCS1 isolate.

## Materials and Methods

### Isolation and Identification of Endophytic Isolate

#### Isolation of Endophyte From Tapobon Forest Patches

Healthy plants of *Lycopodium clavatum* L. were collected from Tapobon forest of Paschim Medinipur district, West Bengal, India (Latitude 22°25^/^ to 22°57^/^N, longitude 87°11^/^E, altitude 23 m above sea level) and endophytic fungi were isolated from stem portion of this pteridophytic plant. Plant body was thoroughly washed by running tap water for 5 min, sodium hypochlorite (2–10%) for 2 min, and hydrogen peroxide (2%) for 1 min, respectively, and explants were incubated on water agar plates at 27°C on biological oxygen demand incubator for endophyte isolation. Water agar plates were supplemented with antibiotics (streptomycin-50 mg/lit and tetracycline-50 mg/lit) to avoid the isolation of bacterial endophytes. The effectiveness of this sterilization and isolation process was cross checked by the explant imprintation technique described by Schulz et al. (1993). In brief, the aliquots used for explant sterilization were spread on water agar medium and incubated under same conditions. After, 3–5 days of incubation, fungal hyphae emerged out from the tissues and they were transferred to PDA plates for optimum growth (Schulz et al., 1993).

#### DNA Extraction and Amplification

Genomic DNA was extracted from 100 mg of fungal hyphal tissue according to the modified procedures of Landum et al. (2016). DNeasy Plant Minikit (Qiagen, Germany) was used according to the manufacturer's instructions. In brief, the first 20 mg of lyophilized fungal samples were disrupted using a mortar and pestle and 400 μl of AP1 buffer, and 4 μl of RNase were mixed well with cell lysate and incubated for a duration of 10 min at 65°C temperature. Next, 130 μl of P3 buffer was added to it and incubated for 5 min on ice. Further cell lysate was centrifuged for 5 min at 20,000 g and 1.5 volumes of AW1 buffer was added to it. After that, 650 μl of the mixture was transferred to DNeasy Mini Spin Column which was placed in a 2 ml collection tube. Centrifugation was done at 6,000 g for 1 min. This process was repeated again and again. The spin column was placed into a new 2 ml collection tube and 500 μl of AW2 buffer was added followed by the centrifugation at 6,000 g for 1 min. Now, 500 μl of AW2 buffer was added and centrifuged for 2 min at 20,000 g. Finally, the spin column was transferred to a new 1.5 or 2 ml microcentrifuge tube. One hundred microliters of AE buffer was used as an eluting solution and the whole mixture was incubated for 5 min at room temperature and then centrifuged for 1 min at 6,000 g. The last step was repeated again and again. Finally, the extracted DNA was subjected to amplification.

The nuclear ribosomal DNA internal transcribed spacer (ITS) of the fungal isolates were amplified using the two universal primer ITS1-Forward primer-5'-TCCGTAGGTGAACCTGCGG-3' and ITS2-Reverse primer-5'- TCCTCCGCTTA TTGATATGC-3' (White et al., 1990). The 25 μl of reaction volume contained 12.5 μl of 2X PCRBio Taq Mix Red (PCR Biosystems, UK) 0.4 μM of both the forward and reverse primers, and 10 ng of genomic DNA template. Another set was produced and termed as negative control where DNA was replaced with distilled water to assure the contamination free reaction. The PCR (Bio-Rad, USA) program configuration was as follows, 1 min at 95°C, 35 cycles at 95?C for 15 s, then again for 15 s at 55°C, 1 min at 72°C, and, finally, 10 min extension at 72°C. Ultimately, the PCR products were separated using 1% agarose gel in 1X TAE buffer (90 mM Tris-acetate and 2nM EDTA, pH 8.0) and stained with ethidium bromide (0.5 μg/ml), before finally documented using BIO-RAD Gel Doc EZ imager version 5.1 (USA). PCR products were sent for direct bi-directional sequencing using ABI 3730xl Genetic Analyzer (Applied Biosystems, USA) to Bioserve Biotechnologies (India) Pvt. Ltd., A Repro Cell Company, Hyderabad, India.

#### Phylogeny Based Identification of the Isolate

rRNA-based molecular identification was adopted for fungal identification (Mahapatra and Banerjee, 2012). Partial sequencing of small and large subunit of ribosomal RNA gene along with complete sequence of ITS1, ITS2, and 5.8S ribosomal RNA gene were done. Further analysis was done using the 616-base pair of consensus sequence. Sequences were submitted to GenBank and compared with pre-existing database through basic local alignment search tool (BLAST). Thirteen sequences, including LCS1, were selected and aligned using multiple alignment software program Clustal W and the phylogenetic tree was constructed using MEGA 7 (Tamura et al., 2007).

### Production and Optimization of EPS

#### Optimization Using One Variable at a Time Method

Endophytic fungi were grown in 250 ml Erlenmeyer flask with 50 ml potato dextrose broth (PDB) in shaker incubator at 120 rpm for 7 days. The pH and temperature of the culture medium was maintained at 6 and 28°C, respectively. In order to optimize the fermentation conditions for endophyte growth in terms of mycelial growth and exopolysaccharide production, varying fermentation time (3–7 days), different medium pH (4.0–8.0), and varying incubation temperature (20–30°C) were tested in separate Erlenmeyer flasks with separate PDB medium. In order to obtain vigorous production, investigation was carried out using various carbon sources (4 g%, w/v of fructose, glucose, maltose, and starch), with different organic and in-organic nitrogen sources (0.3 g% w/v of tryptone, ammonium nitrate, urea, and yeast extract) in different Erlenmeyer flasks with PDB as the common growth medium. After finalization of the additional carbon and nitrogen sources, their optimum concentration was confirmed by applying different concentrations of these products on PDB medium, and the respective biomass and exopolysaccharide amount was calculated. A variety of ionic salts (0.05 g%, w/v of MgCl_2_, FeCl_3_, KCl, and NaCl) were used as a supplementary component with in the medium to assess their ability regarding amplification of fungal biomass and exopolysaccharide. Availability of oxygen was also optimized by growing the fungal hyphae in different medium volume separately in different Erlenmeyer flasks, and parameters like total volume of the flask, medium volume and medium depth, headspace volume, and EPS production surface area were compared in order to provide the optimized conclusion (Wonglumsom et al., 2000). In order to increase the fast availability of oxygen, O_2_ vectors such as liquid alkanes-n-heptane, n-hexane, and n-hexadecane was used in varying concentrations (2–8 g/lit) and the most optimum O_2_ vector was selected for optimum exopolysaccharide production (n-hexane). The best O_2_ vector was added at varying times (0–36 h of the fermentation time) in order to obtain the suitable time period for the addition of liquid alkane for upscaled production.

#### Optimization by RSM (Response Surface Methodology) Using BBD (Box–Behnken Design)

Six major factors, namely, fermentation time, fermentation temperature, medium pH, glucose concentration, yeast extract concentration, and NaCl concentrations, were considered necessary for the maximum production of exopolysaccharides. But the four most major factors (fermentation time, medium pH, glucose concentration, and yeast extract concentration) were considered for RSM studies. The investigational design adopted was a Box–Behnken experimental design with four factors taken from one variable at a time outcome. The design deals with four independent factors each at three different levels of −1.0 (lower than the optimum), 0 (at the optimum value), and +1.0 (higher than the optimum one). Exopolysaccharide production was subjected to a second order polynomial equation by using a multiple regression technique. A factual model concerned to the most significant factors was also obtained. The system performance was defined by the subsequent second order polynomial equation: Y = β_0_ + Σ β_i_X_i_ + Σβ_ij_X_i_X_j_ +Σβ_ii_X${}_{\text{i}}^{2}$, where Y was known as the predicted response or dependent variable, x_i_ and x_j_ were here independent factors, β_0_ was represented as the intercept of the regression equation, β_i_ was called as the linear coefficient, β_ii_ was indicated as the quadratic coefficient, and β_ij_ was designated as the interaction coefficient (Mahapatra and Banerjee, 2016).

### Estimation and Characterization of EPS

#### EPS Estimation

The fungal culture extract was centrifuged at 10,000 rpm in order to separate fungal biomass. Mycelial biomass was collected, dried at 55°C for 24 h, and weighed. The supernatant portion was concentrated in a rotary evaporator under low pressure at a temperature of 40°C. Absolute chilled ethanol was added to the concentrated supernatant (5:1 v/v), mixed thoroughly by a glass rod, and kept for 24 h at 4°C temperature. The viscous precipitate was recovered by centrifugation at 10,000 rpm for 10 min. The concentration of sugar in the EPS sample was estimated spectrophotometrically by phenol sulfuric acid method (Dubois et al., 1956) using glucose as standard. Presence of protein in the EPS sample was determined according to the method of Lowry et al. (1951) using bovine serum albumin as standard. The isolated EPS solution was concentrated in rotary evaporator under low pressure at 40?C for further study. For the analysis of monosaccharide composition, viscous exopolysaccharide was desiccated. For determination of glucan profile, a mushroom β glucan kit (Megazyme Institute Wicklow, Ireland) was utilized.

#### Monosaccharide Analysis

The viscous polysaccharide was first desiccated and the dried polysaccharide was subjected to characterization using GC-MS with some pretreatments (Proestos et al., 2006). One hundred milligrams of dried (powdered) exopolysaccharide was added with 1 ml MeOH, 20 μl ribitol (as internal standard) and 20 μl nor-leucine, and the whole mixture was set on a water bath at 70°C for 15 min. Finally, the whole mixture was centrifuged at 10,000 rpm for 5 min. Then, the supernatant was dried and immediately dissolved in 20 μl methoxy-amine HCL and was kept at 37°C for 120 min. Forty microliters of Tri methyl siloxane (TMS) was added and the ultimate 1 μl EPS sample was injected onto GC-MS for monosaccharide analysis. The instrument was configured with a DB-5 Ultra Inert column (30 m × 0.25 mm). The inlet temperature was 230°C with a split ratio of 25:1 and MS transfer line temperature was set to beat 250°C. Column flow was 1.3 ml/min in a constant flow mode with an average linear initial velocity of 39 cm/sec. ZB-1701 was the guard column with helium as the carrier gas. The program was isothermal with a hold of 5 min at 70°C, then with 10°C/min increase. Eventually, 180°C was reached was held for 2 min. Again, with 10°C/min ramping, temperature was raised to 220°C and held for 1 min. After that, a 2.5°C/min ramp was applied up to 265°C and was then held for 1 min. This was followed by a 10°C/min ramp up to 285°C which was held for 1 min. Lastly, an increase of 1°C/min up to 290°C, held for 0.6 min was given. Detector was in positive ion mode and mass spectrum was acquired in a scan mode from 40 to 650 amu with a detection threshold of 100 ion counts.

### EPS Mediated Plant Growth Promotion

Initially, healthy and disease-free rice seeds (*Oryza sativa* ssp. *indica* MTU-7093 Swarna) were surface sterilized with a series of surface disinfectants: sodium hypochlorite (2.5%) for 20 min, deionized double-distilled water (3–5 times thoroughly), and then soaked in water for germination followed by storing at 22°C for 72–96 h. Uniformly, seeds were transferred to hydroponics box supplemented with Hoagland solution and was replaced at an interval of 3–4 days (Chen et al., 2011). Seedlings reaching an age of 15–20 days were divided into four separate groups with a control group (un-inoculated water), 20, 50, and 100 ppm exopolysaccharides application. Simultaneously, 20% polyethylene glycol (PEG)-6000 (for 7 days) was mixed with hydroponic solution as a drought inducing component, and all the biochemical tests were performed from drought induced seedlings. Further, the fresh weight of rice seedlings was measured and leaves were stored in −20°C for further biochemical estimations.

Relative water content (RWC) of the treated and controlled plants was calculated in percentage following the method of Arndt et al. (2015). Fresh leaves were plucked, and fresh tissue weight was measured, then immersed in a 50-ml tube with distilled water and placed in dark at 4°C for 20 h. Further, the leaves were dried with filter paper and, again, weighed for turgid weight calculation. Lastly, the same leaves were incubated at 80°C for a period of 72 h and dry weight was measured immediately.

Relative water content was calculated by the formula RWC(%) = (FW–DW)/(TW–DW).

Chlorophyll content (mg/g of fresh weight) of the fresh leaves were measured according to the modified formula of Lichtenthaler and Welburn (1983). Firstly, fresh leaves (0.5 g fresh weight) were split into small pieces and immediately dissolved in 50 ml methanol (80% v/v) and covered with black paper or kept in dark conditions for 24–36 h at normal 28-−30°C. Centrifugation was performed and supernatant was estimated (645 and 653 nm) for chlorophyll contents. Chlorophyll content (mg/L FW) = 8.05A_653_ + 20.29A_645_.

In order to calculate the proline contents of the seedling, method proposed by Bates et al. (1973) was adopted. Leaves (0.5 g, fresh weight) were split into small pieces and were put in a test tube. Further treatment was done by mixing with 5 ml of 3% sulfosalicylic acid, incubated in water bath for 10 min, and 2 ml of the supernatant was mixed thoroughly with 2 ml of acetic acid and 3 ml of 2.5% ninhydrin. Finally, the mixture was incubated in a water bath for a time period of 40 min - 1 h and was extracted using 4 ml methylbenzene. Optical density was measured in 520 nm and was compared with a proline standard curve.

Regarding the estimation of soluble sugar content (in terms of mg/g fresh weight), the popular method of Watanabe et al. (2000) was followed. Fresh leaves (0.2 g, fresh weight) were crushed in 80% v/v ethanol (10 ml) and were centrifuged at 8,000 g for 10 min at 4°C. One milliliter of supernatant was thoroughly mixed with 3 ml of anthrone reagent followed by high heating at 100°C for 10–12 min and was stopped by rapid cooling on the ice. Finally, a 620 nm absorbance was estimated using glucose as a standard.

Malondialdehyde content (μmol/L fresh weight) was reported according to the method of Del Buono et al. (2011). Zero point five grams of fresh leaves were homogenized in 5% (w/v) trichloroacetic acid (TCA) and centrifuged at 12,000 g for a time period of ~15–20 min. The supernatant was mixed with 5 ml of 0.5% thio-barbituric acid (TBA) prepared with 20% TCA followed by incubation for 25 min before cooling at 100°C. Now, the mixture is left for cooling. Finally, after centrifugation (7,500 g for 5 min), the supernatant was measured for its absorbance at 450, 532, and 600 nm. Amount of malondialdehyde (MDA) was calculated by the following formula, MDA content (μmol/ L) = 6.45 (A_532_-A_600_)-0.56A_450_.

The methods of Lei et al. (2015) were followed in the case of peroxidase components measurement. The whole system contained addition of variety of chemicals, 2.9 ml of 0.05 M phosphate buffer, 0.5 ml of 2% H_2_O_2_, 0.1 ml of 2% guaiacol, and 0.1 ml of crude enzyme extract followed by the absorbance measurement at 470 nm. Lastly, peroxidase (POD) activity was calculated as an amount of guaiacol oxidized per minute in nanomoles per minute per mg of protein. At the end of the reaction, the absorbance was measured at 470 nm. POD activity was defined as the amount of guaiacol oxidized per minute, and was expressed as nanomoles per minute per mg of protein.

Catalase activity was calculated following the protocols of Lei et al. (2015): 0.1 ml H_2_O_2_ (2%) and 2 ml phosphate buffer (50 mM-pH 7.0) were mixed and the whole reaction was initiated by the addition of 0.1 ml of crude enzyme extract. Finally, the catalase activity was measured (at 240 nm) in terms of decrease of values of H_2_O_2_ per minute, as nanomoles/minute/gm of protein.

Superoxide dismutase activity was assayed following the protocols of Lei et al. (2015). The whole system contained a series of valuable freshly prepared reagents: 1.5 ml of 0.05 M phosphate buffer 0.3 ml of 130 mM methionine solution 0.3 ml of 750 μM nitroblue tetrazolium solution 0.3 ml of 100 μM EDTA -Na_2_ solution 0.3 ml of 20 μM lactochrome solution 0.5 ml of distilled water, and finally 0.1 ml of crude enzyme extract (plant source-leaf). The complete reaction was initiated at 4,000 Lx of illumination for constant 20 min with no interruption. The control set comprised of same set of reagents and illumination but with no crude enzyme extract, rather replaced with a phosphate buffer. The third set up of control contained only phosphate buffer followed by incubation in dark conditions for the same time period of 20 min. Finally, after the completion of reaction, the absorbance was estimated at 560 nm of wavelength. One unit of superoxide dismutase (SOD) activity was defined as the amount of enzyme which inhibits NBT reduction by 50%. Also, the results were expressed as unit/mg protein.

### Statistical Analysis

All experiments were performed in triplicate and the results were presented as means ± standard errors (SE). Data were analyzed by Prism GraphPad software version 9.2.0 (332) (San Diego, California, USA). Minitab (version 20.2) statistical software was used for Response surface methodology experiments (Box-Behnken Design).

## Results

### Identification of the Fungal Isolate

In total six endophytic fungi were isolated from the explants, but only one isolate was selected for this study due to its supremacy in exopolysaccharide production. No bacterial isolate was found as antibiotics were previously supplemented with in the isolation media. The water agar plates where the sterilization solution was poured lack any endophytic fungi, thus, proves the true endophytic nature and also non-epi-phytic nature of the isolate. As disease-free plants were selected for endophyte isolation, the chance of pathogenic fungi isolation was also eliminated.

**Figure 1:**
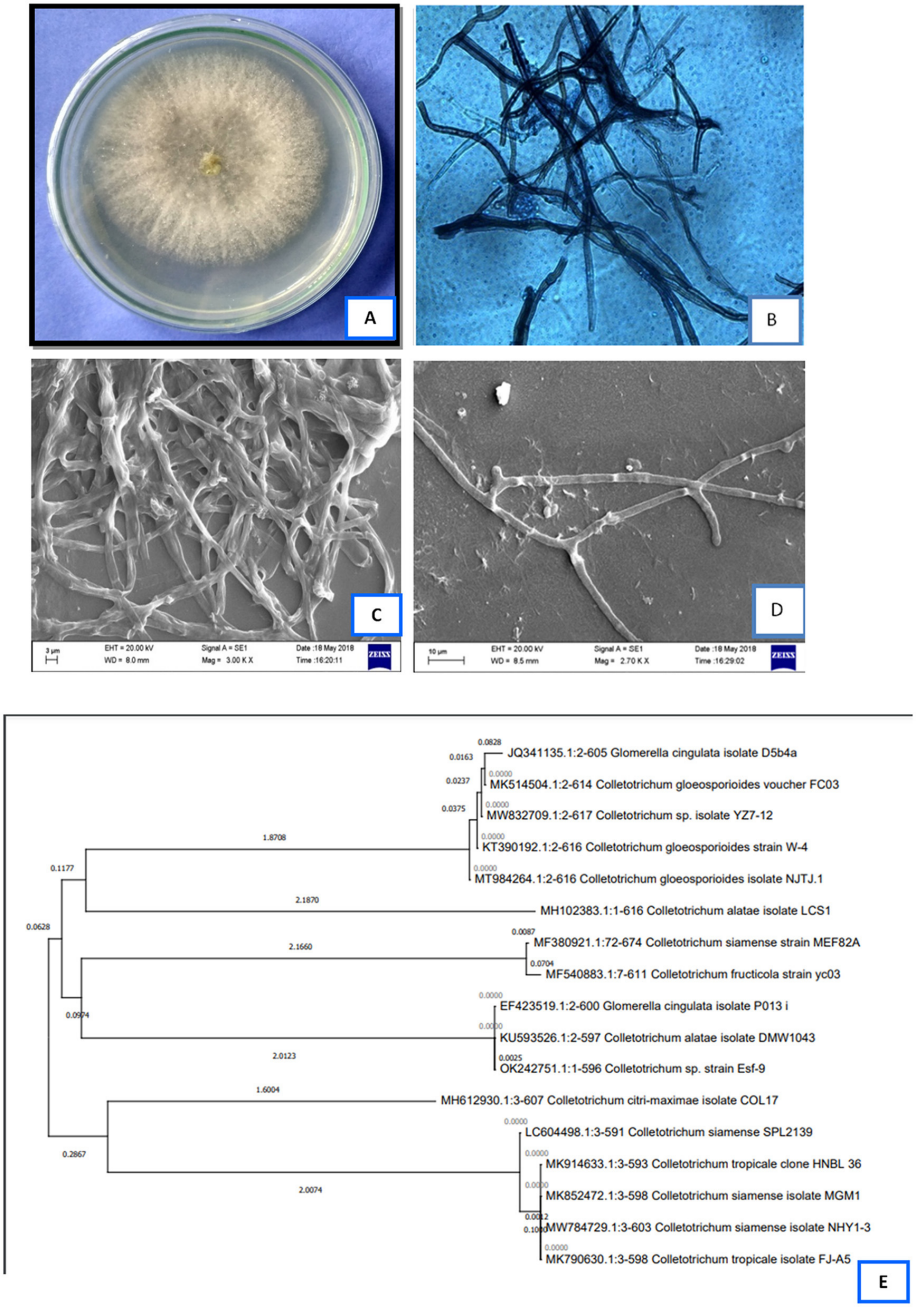
**(A)**-plate morphology with top view, **(B)**-light microscopic photograph of the isolate, **(C,D)**-scanning electron microscopic image of the sterile hyphae, **(E)**-phylogenetic analysis of the endophytic *Colletotrichum alatae* LCS1.

The fungal isolate did not produce any reproductive structure and was identified as *Colletotrichum alatae* LCS1 (GenBank Acc. No.-MH102383.1). A phylogenetic tree was constructed using ITS 1/5.8S rDNA/ITS 2 sequences using MEGA 4 software. The constructed phylogenetic tree provides information about the identity of the endophytic fungi studied for its bioactivity (Figure 1E). Micro-photographs are taken using light and scanning microscopic system showing sterile mycelial structure (Figures 1B–D) along with the plate morphology with top and bottom view (Figure 1A).

### Optimization of Necessary Fermentation Parameters

#### Optimization by OVAT Method

*Colletotrichum* sp. LCS1 was grown on 250 ml Erlenmeyer flask in submerged condition for 8 days. The day wise variation of fungal growth and respective exopolysaccharide production is presented in Table 1. It was found that there was a parallel response of fermentation time on biomass production and exopolysaccharide yield. Up to day 5, both biomass and exopolysaccharide yield got enhanced simultaneously, but from the 6th day (EPS yield 9.67 ± 0.58 g/l), both the values fall slowly. There was a fall on both the values up to 10th day (Table 1).

**Table 1:** Effect of various physical parameters and chemical supplements on biomass and exopolysaccharide (EPS) production by *Colletotrichum alatae* LCS1.

**Parameters**	**Effectors**	**Percentage added (g%)**	**Biomass (g/l)**	**Exopolysaccharide production (gm/lit)**
Incubation time (in day)	3^rd^ day	**-**	3.566 ± 0.057	8.33 ± 0.64
	4^th^ day	**-**	4.466 ± 0.115	10.13 ± 0.58
	5^th^ day	**-**	5.343 ± 0.045	11 ± 0
	6^th^ day	**-**	4.916 ± 0.020	9.33 ± 0.13
	7^th^day	**-**	4.133 ± 0.057	7 ± 1
Incubation temperature (°C)	22	**-**	5.970 ± 0.026	8 ± 0
	24	**-**	6.756 ± 0.049	9.33 ± 0.18
	26	**-**	7.863 ± 0.030	12.13 ± 0.25
	28	**-**	4.963 ± 0.046	9.67 ± 0.43
	30	**-**	3.076 ± 0.015	7 ± 0
Initial medium pH	4	**-**	3.906 ± 0.010	7.33 ± 0.24
	5	**-**	6.012 ± 0.011	10.03 ± 0.19
	6	**-**	7.896 ± 0.015	13.03 ± 0.14
	7	**-**	7.033 ± 0.049	8 ± 0
	8	**-**	6.156 ± 0.049	5.33 ± 0.19
Additional carbon source	Starch	3	7.877 ± 0.001	8.67 ± 0.28
	Fructose	3	7.167 ± 0.017	11.67 ± 0.15
	Glucose	3	8.063 ± 0.054	14.00 ± 0.10
	Maltose	3	7.084 ± 0.018	10 ± 1
Additional nitrogen sources	Tryptone	0.2	8.073 ± 0.004	9.33 ± 0.58
	Yeast extract	0.2	8.43 ± 0.010	15.01 ± 0.01
	NH_4_NO_3_	0.2	8.066 ± 0.005	8 ± 0.06
	Urea	0.2	8.19 ± 0.076	12.56 ± 0.87
Glucose concentration	Glucose	3	8.423 ± 0.004	14.16 ± 0.10
		5	8.743 ± 0.004	14.89 ± 0
		7	9.742 ± 0.004	14.99 ± 0.08
		9	9.02 ± 0.001	14 ± 0
		11	8.866 ± 0.005	11.33 ± 0.58
Yeast extract concentration	0.2	-	8.410 ± 0.010	8.67 ± 0.10
	0.4	-	9.065 ± 0.011	11.33 ± 0.28
	0.6	-	10.713 ± 0.004	15.50 ± 0.08
	0.8	-	8.113 ± 0.004	13.67 ± 0.37
	0.10	-	7.205 ± 0.004	12.33 ± 0.11
Different metal ions	NaCl	0.50	10.134 ± 0.056	15.60
	KCl	0.50	7.011 ± 0.006	8.67 ± 0.55
	MgCl_2_	0.50	5.706 ± 0.009	9.33 ± 0.85
	CaCl_2_	0.50	3.011 ± 0.006	10.33 ± 0.81
NaCl concentration		0.50	7.952 ± 0.063	11.33 ± 0.0.78
		0.1	9.069 ± 0.043	15.69 ± 0.09
		0.2	8.006 ± 0.004	13.67 ± 0.67
		0.3	7.069 ± 0.043	12 ± 1

Fungal growth and EPS production were studied in a temperature range of 22–30°C (Table 1). The maximum EPS yield and biomass was obtained at 26°C (EPS g/l). An increase or decrease from this temperature value causes a sharp decline in EPS production and in biomass production. The reason of such negative relationship after a particular range between the biomass estimation and exopolysaccharide production was due to inactivation of some particular enzymes needed for exopolysaccharide production (Mahapatra and Banerjee, 2013).

Exopolysaccharide (EPS) yield related to fungal growth was measured at variable pH conditions of the medium (4–8). The optimum EPS and biomass production was found at the slightly acidic pH of 6 (Table 1). A slight change from this value deviates the optimum biomass production slightly but hampers EPS production drastically. This is due to inhibition of fungal metabolic pathways at high acidic or basic condition that leads to inactivation of necessary enzymes along with the blocking of several intrinsic pathways (Mahapatra and Banerjee, 2009).

Fungi exhibits maximum preferences toward carbohydrates as a reliable carbon source for optimum growth and metabolic activity. The EPS production was evaluated with a variety of additional carbon sources like glucose, fructose, maltose, and starch. Though there was a slight difference in terms of EPS production in case of the four tested carbohydrate sources, but glucose is comparatively superior and being cost-effective is gracefully selected for optimum activity. This carbohydrate-based growth and metabolism-related expression of the endophytic fungi was due to varied effects of catabolic repression of different sugar sources on exopolysaccharide production and biomass (Nehad and El-Shamy, 2010). Glucose, being the most effective carbon source for EPS production, was tested at different concentrations for maximized yield. A glucose concentration of 2–10 g%lit was tested for EPS production and up to a certain concentration (7 g%), the EPS yield was satisfactorily increasing but after that, the values fall drastically. The effective glucose concentration was found to be 7 g%/lit with an outcome of 14.99 ± 0.08 gm/l EPS.

Nitrogen is another major factor for fungal growth. Organic (yeast extract, urea and tryptone) and inorganic (NH_4_NO_3_) nitrogen sources were tested for the highest outcome. Yeast extract at a percentage of 0.4 g%/lit was found to be the most effective nitrogen source in order to obtain the maximum exopolysaccharide production of 11.33 ± 0.28 gm/l over other organic and inorganic (NH_4_NO_3_) nitrogen sources. The preference of yeast extract over other nitrogen sources was due to shifting of metabolic pathways and synthesis of altered types of metabolites in the presence of yeast extract, which promotes fungal growth (Banerjee and Mahapatra, 2009).

Metal ions in the form of additional salt in fermentation medium is necessary for physiological functioning of a fungal cell. So, the EPS production can also be altered according to the type and concentration of metal ion availability. Salts are generally known to influence the growth and metabolism of fungal strains. Metal ions act as catalysts or co-factors for the function of several key enzymes which are needed for production of growth necessary compounds. In this study NaCl (conc. −0.10 g%/lit) supplemented media showed maximum EPS yield in comparison to KCl and MgCl_2_, but after a certain level reaching the optimum position, the values rapidly decreased (Table 1). Actually, the metal ions modify the cell membrane permeability and promotes easy and bulk excretions of EPS. A decrease in EPS amount was due to accumulation of excess amount of metal ion, leading to toxicity in the culture medium (Hwang et al., 2003).

Oxygen availability is one of the prime factors for optimum growth of fungal cells and production of exopolysaccharides. Erlenmeyer flask, being a closed system for fungal fermentation, contains a limited availability of oxygen. So, there is a contrasting relationship between medium volume and oxygen availability. Also, higher medium volume provides a lower surface area for O_2_ interaction. But, the head-space volume and O_2_ availability has a strong positive relation. The details of O_2_ availability to obtain the maximized production is summarized in Table 2. Finally, a 50 ml of fermentation medium was found to be the best suited for fungal growth and exopolysaccharide production.

**Table 2:** Effect of different medium volume on dissolved oxygen (DO) parameters in fermentation system with respective biomass and EPS production by *Colletotrichum alatae* LCS1.

**Medium volume (ml)**	**Total volume of flask (ml)**	**Head space volume (ml)**	**Medium depth (cm)**	**Surface area (cm)**	**Biomass (g/l)**	**EPS yield (gm/lit)**
25	320	295	1	2.79	4.763 ± 0.215	6.33 ± 0.08
50	320	270	1.5	2.65	9.777 ± 0.091	15.67 ± 0.19
75	320	245	2	2.53	5.112 ± 0.415	12.33 ± 0.21
100	320	220	2.5	2.39	5.447 ± 0.819	11.33 ± 0.28

Other than the headspace volume and surface area for O_2_ interaction, availability of oxygen can also be enhanced by O_2_ vectors. Out of three O_2_ vectors (n-hexane, n-hexadecane, and n-heptane), n-hexane was found to be the most efficient one at a concentration of 4 g%/lit for fungal growth and exopolysaccharide production, but n-heptane and n-hexadecane caused decrease in fungal biomass but provided a slight enhancement in exopolysaccharide output. Also, the addition time of this vector (n-hexane) to the fermentation medium can influence the fungal growth and exopolysaccharide production. It was found that adding of liquid alkane at the initial stage of fermentation is the best way to have higher yield. When the vector is added at 4, 6, and 8 h, there is no such significant difference in EPS yield than the 0 h addition. But addition during mid time or end stage of fermentation exhibits detrimental effect on both the biomass production and EPS yield (Table 3).

**Table 3:** Effect of oxygen vectors (n-hexane, n-hexadecane, n-heptane) on EPS yield.

**Time of addition (hours)**	**n-hexane**		**n-heptane**		**n-heptadecane**	
	**EPS (g/L)**	**Biomass (g/L)**	**EPS (g/L)**	**Biomass (g/L)**	**EPS (g/L)**	**Biomass (g/L)**
0	17.38 ± 0.21	10.01 ± 0.01	15 ± 0.45	08	12.19 ± 0.98	07.19 ± 0.87
6	15.96 ± 0.12	09.23 ± 0.12	13.09 ± 0.04	07.65 ± 0.04	11.78 ± 0.09	06.07 ± 0.97
12	13.67 ± 0.67	08.10 ± 0.10	12.98 ± 0.01	07.00 ± 0.01	10.08 ± 0.01	05.78 ± 0.61
18	12.81 ± 0.19	08.00 ± 00	11.00 ± 0.70	06.09 ± 0.12	09.87 ± 0.02	04.99 ± 0.07
24	10.97 ± 0.23	07.19 ± 0.19	08.98 ± 0.12	05.10 ± 0.19	08.00 ± 0.91	04.00 ± 0.19
30	08.12 ± 0.11	06.17 ± 0.23	07.12 ± 0.27	05.00 ± 0.01	07.87 ± 0.007	03.78 ± 0.04
36	07.98 ± 0.17	06	06.13 ± 0.09	04.09 ± 86	07	03.00 ± 0.01

#### Optimization by Box-Behnken Design

One variable at a time (OVAT) methods have some limitations regarding the best suitable data interpretation among the different interacting factors but when it is coupled with Response surface methodology (RSM), it can minimize the process and hard work generally interpreting the interaction between different factors. It not only reduces the effort but also provides a statistically significant model for proper optimization of fermentation procedures. Here, a three level Box-Behnken design, including four factors (Glucose concentration, yeast extract concentration, pH of medium, and fermentation time) with five replicates at the center point of each factor, was introduced as a model for analysis of exopolysaccharide production. The experimental values along with the predicted values by the statistical system are represented in Table 4. There was a variation in exopolysaccharide production according to the variation of fermentation conditions. The replicated center points (five) always represented the highest values for exopolysaccharide production. The predicted response (Y) for the production of exopolysaccharides (gm/lit) by the endophytic isolate *Colletotrichum alatae* LCS1 based on the coded fermentation factors is represented as the following equation: Y_EPS(gm/lit)_ = −494.97 + 78.46 X_1_ + 8.959 X_2_ + 136.09 M X_3_ + 8.961 X_4_ – 78.996 X${}_{1}^{2}$ – 0.69777 X${}_{2}^{2}$ – 10.459 X${}_{3}^{2}$ – 0.95402 X${}_{4}^{2}$ – 1.3125 X_1_X_2_ + 0.050 X_1_X_3_+ 0.9687 X_1_X_4_ + 0.0575 X_2_X_3_ + 0.28938 X_2_X_4_ – 0.1500 X_3_X_4_. Here, Y_(EPSgm/lit)_ was the predicted exopolysaccharide yield and X_1_, X_2_, X_3_, and X_4_ were coded factors of yeast extract concentrations, glucose concentrations, medium pH and fermentation time, respectively.

**Table 4:** Experimental design with predicted and measured outcome of EPS yield using Box–Behnken design regarding *C*. *alatae* LCS1 fermentation system.

**Run**	**Independent variables**				**Response**	
	**X1: YEC (g%)**	**X2: GC (g%)**	**X3: M pH**	**X4: FT (day)**	**Measured**	**Predicted**
1	0 (0.4)	0 (7)	0 (6)	0 (5)	15.69	15.98
2	1 (0.5)	0	1 (7)	0	8.5100	8.5600
3	0	0	−1 (5)	−1 (4)	11.3900	11.3529
4	0	1 (8)	−1	0	8.5700	8.6404
5	−1 (0.3)	0	0	1 (6)	7.6800	7.7471
6	0	−1 (6)	1	0	12.3500	12.3488
7	0	1	0	1	7.2000	7.1783
8	0	0	−1	1	8.0400	8.9996
9	−1	0	1	0	11.5400	11.5083
10	0	1	0	−1	7.5500	7.5167
11	0	0	0	0	15.61	15.98
12	−1	−1	0	0	12.5000	12.4646
13	0	0	1	1	7.4500	7.4013
14	0	1	1	0	8.3800	8.4571
15	1	0	0	1	6.5700	6.5638
16	0	−1	−1	0	11.7700	11.7621
17	1	0	0	−1	8.4400	8.4421
18	1	−1	0	0	11.6000	11.5563
19	0	0	1	−1	5.4000	11.3546
20	−1	0	−1	0	11.8500	11.8167
21	0	0	0	0	15.63	15.98
22	1	0	−1	0	5.8000	5.8483
23	−1	1	0	0	9.5500	9.5079
24	0	−1	0	−1	12.8000	12.8383
25	−1	0	0	−1	11.1000	18.1754
26	1	1	0	0	6.5500	6.4996
27	0	−1	0	1	7.8200	7.8700
28	0	0	0	0	15.66	15.98
29	0	0	0	0	15.67	15.98

**Figure 2:**
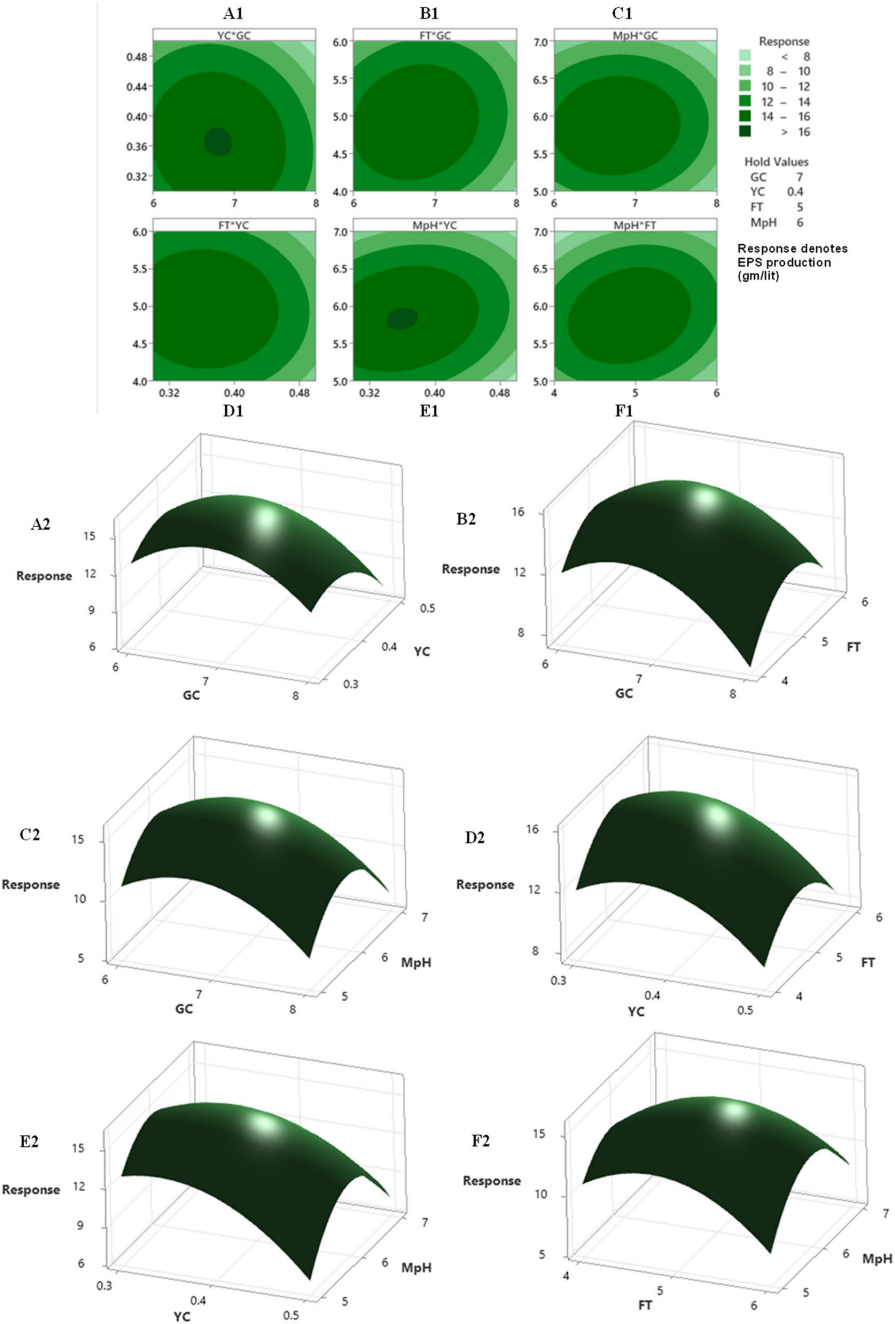
The contour plot and 3D-plot with 2D-projection showing the most important interactions of factors in response surface methodology (RSM) optimization of exopolysaccharide production by *Colletotrichum alatae* LCS1. **(A1,A2)** yeast extract concentration (YC)-0.4 gm/lit. vs. glucose concentration (GC)-7 gm/lit. at fermentation time (FT) 5 days and medium pH (MpH) 6. **(B1,B2)** between FT (5 days) vs. GC (7 gm/lit.) at yeast extract concentration (0.4 gm/lit) and MpH (6). **(C1,C2)** between MpH (6) vs. GC (7 gm/lit.) at YC (0.4 gm/lit.) and FT (5 days). **(D1,D2)** between FT (5 days) vs. YC (0.4 gm/lit.) at GC (7 gm/lit.) and MpH (6). **(E1,E2)** between MpH(6) vs. YC (0.4 gm/lit.) at GC (7 gm/lit.) and FT (5 days). **(F1,F2)** between MpH vs. FT (5 days) at GC (7 gm/lit.) and YC (0.4 gm/lit.).

A regression analysis was recommended for checking the goodness of fit of the RSM with experimental output data. The F test data having a large value of 3,507.30 indicated that the model was significant. There is no chance that this type of large F value could originate due to noise issues. The adjusted determinant coefficient ($R_{\text{adj}}^{2}$) of the model help in evaluation of the goodness-of-fit of the regression equation. $R_{\text{adj}}^{2}$ value was found to be 0.9994, which denotes that there was a high degree of correlation between the experimental and predicted value. It could be concluded that the fitness of the model was good as it has the lack of fit F value of 1.10, which was not at all significant related to the obtained pure error. The model *P* < 0.0001 focus on the fact that the model equation was appropriate regarding the exo-polysaccharide production. The *P*-value for lack of fit for this polynomial equation was 0.505, which is higher than 0.05 that indicates the accuracy of this system. The linear and quadratic effects of glucose concentration, yeast extract concentration, fermentation time, and pH of fermentation medium were significant (*P* < 0.0001) in this model (Table 5). The interactions among the four different variables regarding the production of exopolysaccharide were analyzed. The interaction where *P* < 0.0001, they have positive effects, whereas in the interaction between medium pH and glucose concentration, medium pH and yeast extract concentration (*P* > 0.0001) were of least importance as the *P* > 0.0001. Response surface plots were constructed using contour plotting through the help of Minitab (20.2). The contour plots focused on the portions where the best interactions had been made between the different interacting factors to obtain the optimum results (Figure 2). The model assumed a maximum response for exopolysaccharide production in terms of 15.98 gm/lit when the four interacting factors will be yeast extract concentration (YEC) 0.47 g%, glucose concentration (GC) 7.53 g%, M-pH (medium pH) 6.46, and fermentation time (FT) 5.61. The predictions were performed in laboratory to evaluate its success and it had been found that following the parameters *Colletotrichum alatae*, LCS1 yielded a response of 15.69 ± 0.20 gm/lit EPS. Experimental verification established the fitness of the model and, also, the optimum conditions were finally shorted out through the minimum effort.

**Table 5:** ANOVA for response surface quadratic regression model of exopolysaccharide yield of *Colletotrichum alatae* LCS1.

**Source**	**DF**	**Adj SS**	**Adj MS**	***F*-Value**	***P*-Value**
Model	14	232.552	16.6108	3507.30	0.000
Linear	4	60.013	15.0032	3167.86	0.000
YEC (X_1_)	1	11.505	11.5052	2429.27	0.000
GC (X_2_)	1	27.120	27.1201	5726.29	0.000
M pH (X_3_)	1	0.267	0.2670	56.38	0.000
FT (X_4_)	1	21.121	21.1205	4459.50	0.000
Square	4	165.373	41.3433	8729.44	0.000
YEC*YEC (X${}_{1}^{2}$)	1	64.765	64.7646	13674.76	0.000
GC*GC (X${}_{2}^{2}$)	1	50.531	50.5307	10669.32	0.000
M pH*M pH (X${}_{3}^{2}$)	1	44.350	44.3504	9364.39	0.000
FT*FT (X${}_{4}^{2}$)	1	94.459	94.4594	19944.68	0.000
2-Way Interaction	6	7.166	1.1943	252.17	0.000
YEC*GC (X_1_X_2_)	1	1.103	1.1025	232.79	0.000
YEC*M pH (X_1_X_3_)	1	0.000	0.0001	0.02	0.887
YEC*FT (X_1_X_4_)	1	0.601	0.6006	126.82	0.000
GC*M pH (X_2_X_3_)	1	0.013	0.0132	2.79	0.117
GC*FT (X_2_X_4_)	1	5.359	5.3592	1131.58	0.000
M pH*FT (X_3_X_4_)	1	0.090	0.0900	19.00	0.001
Error	14	0.066	0.0047		
Lack-of-Fit	10	0.049	0.0049	1.10	0.505
Pure Error	4	0.018	0.0044		
Total	28				
R-sq		99.97%			
R-sq(adj)		99.94%			
R-sq(pred)		99.87%			

### Biochemical Characterization (Monosaccharide Analysis) of EPS

**Figure 3:**
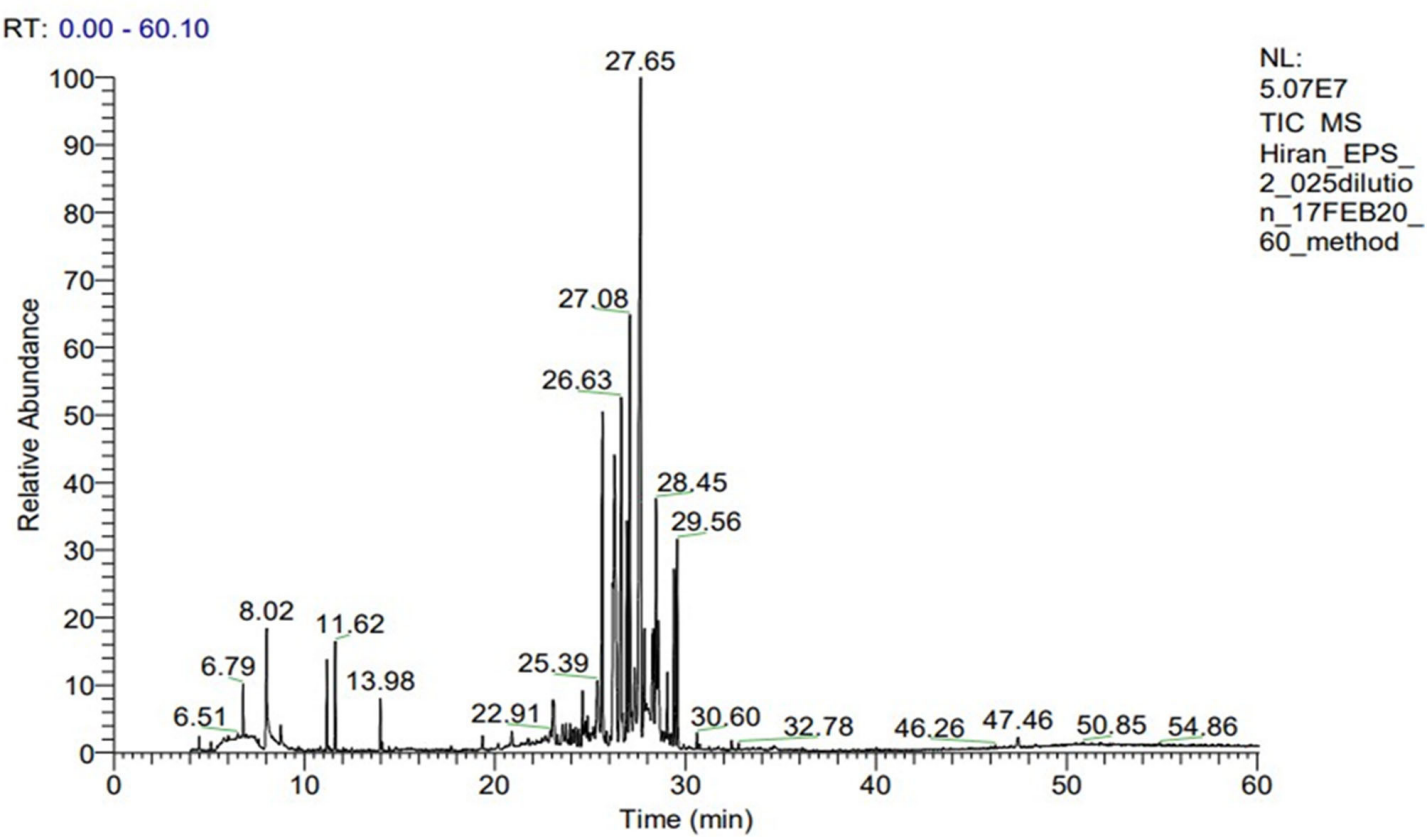
GC-MS spectra of heteropolysaccharide from *C*. *alatae* LCS1 showing monosaccharide compositions at different retention times.

Exopolysaccharide (EPS) was found to be heteropolysaccharide in nature and is enriched of β-glucan. The whole process of monosaccharide analysis was programmed in GC-MS X-Calibur software for 1 h and different monosaccharide components were detected in different retention time (RT) of the column system-glucose [(31.64%)−27.65 min, arabinose (20.25%)−17.08 min, rhamnose (19.62%)−19.62 min, galactose (28.45%)−17.59 min, mannose (16.46%)−25.10 min, and fucose (10.12%)−29.65 min]. The glucose was found to be the most abundant monosaccharide followed by arabinose, rhamnose, galactose, mannose and fucose. Glucan profile of EPS includes the total glucan, α glucan and β glucan as an amount of 32.09 ± 0.31, 12.61 ± 0.30, and 19.48 ± 0.19 g/100 gm of EPS, respectively. The details of the GC-MS chromatogram highlighting the peaks of monosaccharide composition is represented in Figure 3. The monosaccharide components, along with their retention time and area percentage, was presented in Table 6. Though the program was run for 1 h (60 min), all the sugar components were detected within 30 min of initialization. The peaks originated between 2 and 12 min contain the peaks of the solvents and other derivatizing components (methanol, ribitol, norleucine, and TMS in a repetitive manner). The derivatized sugar components showed a prominent peak during 24–30 min, revealing all the monosaccharides.

**Table 6:** Monosaccharide compositions of *C*. *alatae* LCS1 heteropolysaccharide.

**Sl. No**.	**Monosaccharide compositions**	**Retention time (minute)**	**Area%**
1	Mannose	25.10	16.46
2	Rhamnose	26.63	19.62
3	Arabinose	27.08	20.52
4	Glucose	27.65	31.64
5	Galactose	28.45	17.59
6	Fucose	29.56	10.12

### Drought Resistance Activity of Exopolysaccharides

#### Betterment of Physical Characters of the Rice Seedlings

**Figure 4:**
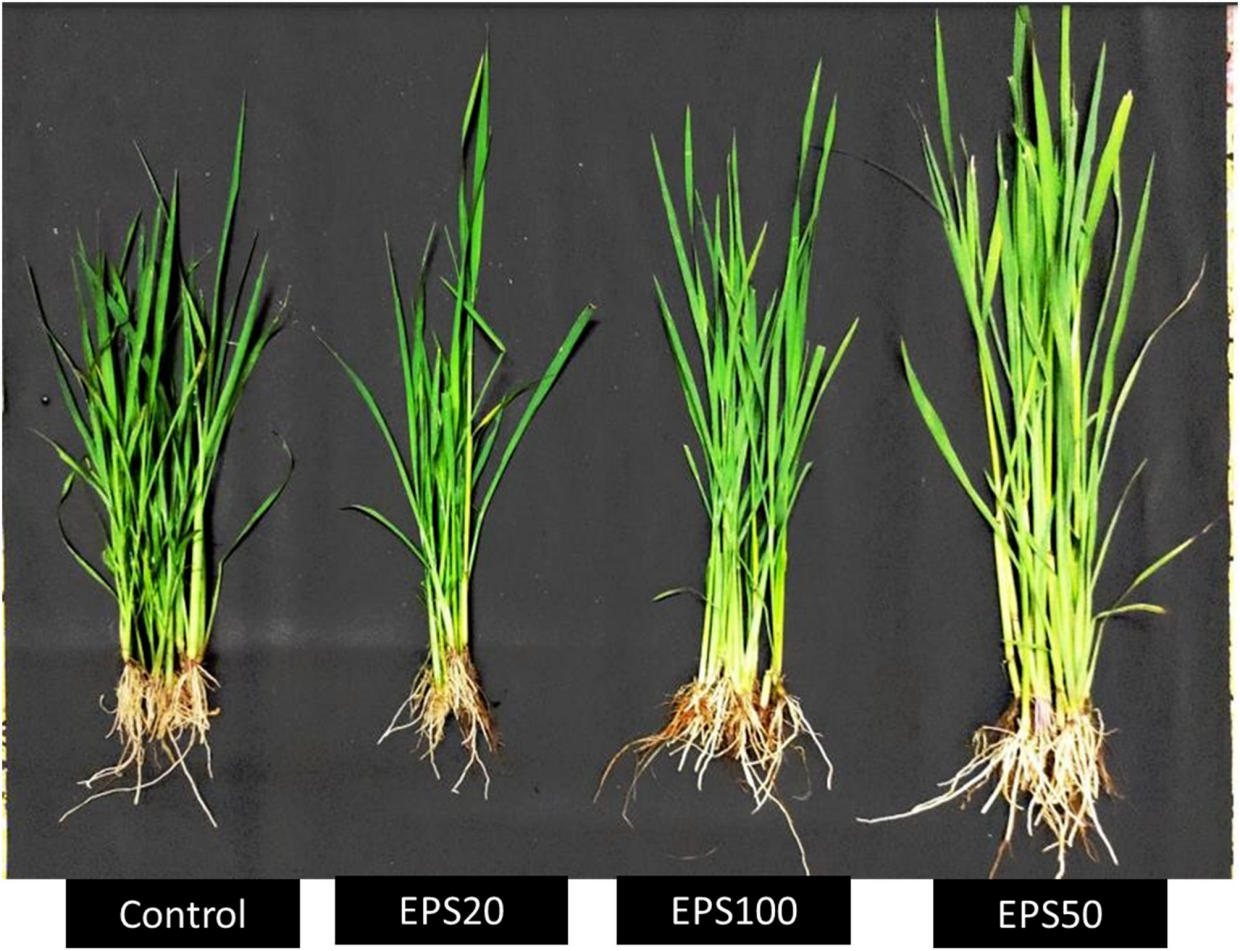
Phenotypes of rice seedlings *Oryza sativa* ssp. *indica* MTU 7093 Swarna under drought stress (induced by PEG treatment) sprayed with 20, 50, and 100 ppm EPS of *C*. *alatae* LCS1 endophyte.

In case of only PEG (drought stress inducer)-treated plants (control set of plants), there was a detrimental effect on the rice seedlings as the leaves become yellow and weight gets decreased. But in case of the seedlings treated with PEG and EPS (20, 50, and 100 ppm) simultaneously, there was a significant improvement of the plant qualities in terms of drought stress alleviation (fresh weight and relative water content gets improved than the control sets). The control comprises of only PEG application whereas the other three groups were treated with PEG along with 20, 50, and 100 ppm EPS application, respectively. The seedlings that were treated with foliar spray EPS (20, 50, and 100 ppm) were found to exhibit higher fresh weight contents. Particularly, 1.13, 1.28, and 1.79 times higher, respectively, than the control one. Relative water content was found to be elevated in case of the foliar sprayed seedlings. Particularly, 28.3, 33.24, and 13.90% increase in case of 20, 50, and 100 ppm foliar spray, respectively, in comparison to control (Table 7). In Figure 4, the four types of plants (control, EPS treated-20 ppm foliar spray, 50 ppm foliar spray, and 100 ppm foliar spray) are presented for clear understanding of the drought stress alleviation potentiality of EPS foliar spray on rice seedlings (*Oryza sativa* ssp. *indica* MTU 7093 Swarna).

**Table 7:** Fresh weight and relative water contents of exopolysaccharide sprayed rice seedlings.

**Treatment group (EPS concentration)**	**Fresh weight (mg)**	**Relative water contents (%)**
Control	118.93 ± 2.375a	85.100 ± 1.010
EPS 20 (20 ppm)	134.39 ± 3.010b	109.48 ± 1.109
EPS 50 (50 ppm)	212.89 ± 5.091c	113.79 ± 1.271
EPS 100 (100 ppm)	152.23 ± 2.091d	96.56 ± 0.989

*One-way ANOVA (Tukey's Multiple Comparison test) was performed to check the potential statistical differences and the treated (20, 50, and 100 ppm EPS foliar spray) plants showed statistically valid differences (P <0.05, the four different letters a, b, c, and d indicates significance differences) from the control plant for the above said parameters*.

#### Improvement of Biochemical Parameters

**Figure 5:**
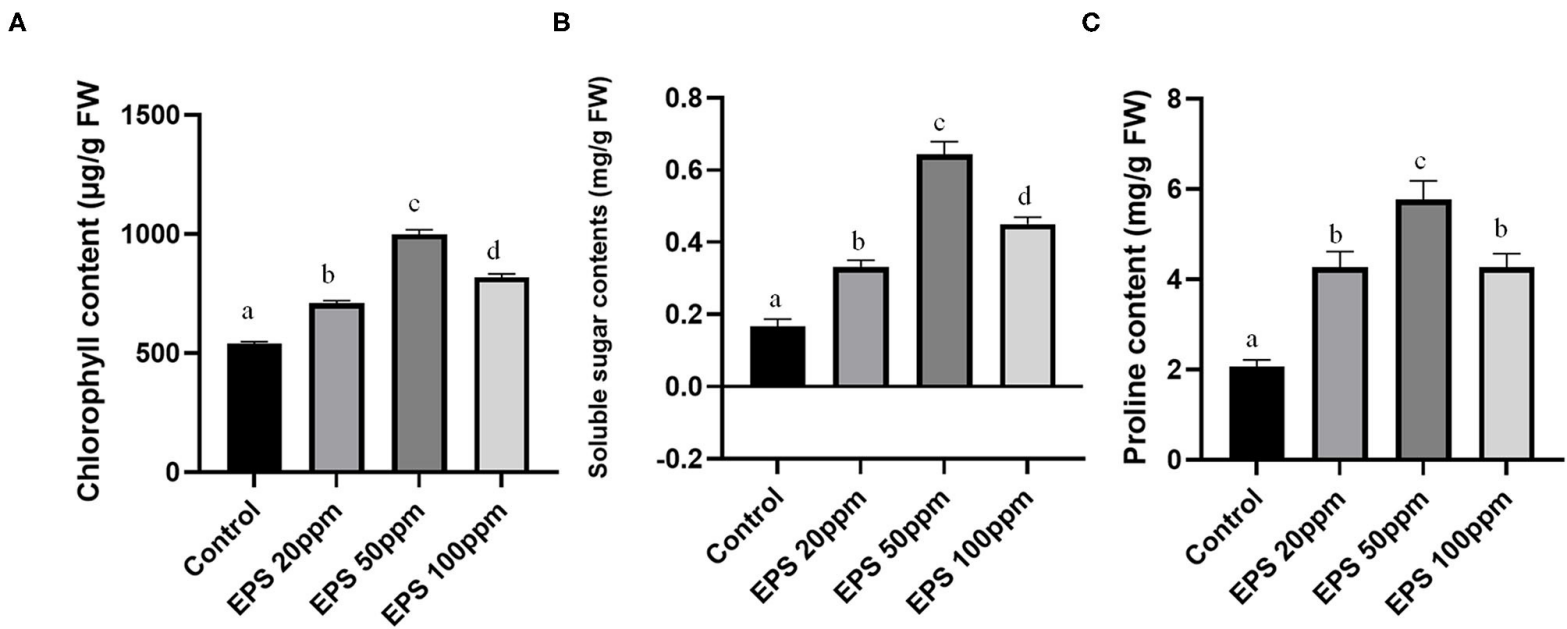
The effect of EPS foliar spray (20, 50, and 100 ppm) on chlorophyll content **(A)**, soluble sugar content **(B)**, and proline content **(C)** on rice seedlings. Values on the graphs are the means ± standard error (SE) of three replicates. Tukey's Multiple comparison test was performed. The different letter a, b, c, d indicates significance differences in comparison to the control plant (At, *P* < 0.05).

Chlorophyll is the most necessary component of plant leaves and necessary for plant survival and optimum growth. The seedlings sprayed with 20, 50, and 100 ppm fungal EPS (along with PEG treatment) showed elevated chlorophyll content (20 ppm- 699.19 μg/g fresh weight, 50 ppm- 979.81 μg/g fresh weight, and 100 ppm-801.90 μg/g fresh weight) with 1.31, 1.84, and 1.51-fold increase in comparison to control plants (532.49 μg/g fresh weight), which were devoid of any EPS treatment (only drought inducer PEG was applied). The graphical representation of chlorophyll contents of different sets of plant is represented in Figure 5A.

Other than chlorophyll content, other biochemical parameters like soluble sugar contents and proline contents also showed higher values (2.6, 4.06, 2.73, 2.05, 2.78, and 2.21-fold increase) in case of 20, 50, and 100 ppm EPS application than the control one (Figures 5B,C). Proline contents usually promote the salt balance and osmotic pressure of the cells, and thus promote drought stress tolerance. Soluble sugars as a whole provide a better nutrient in the plant tissue providing improved vigor.

**Figure 6:**
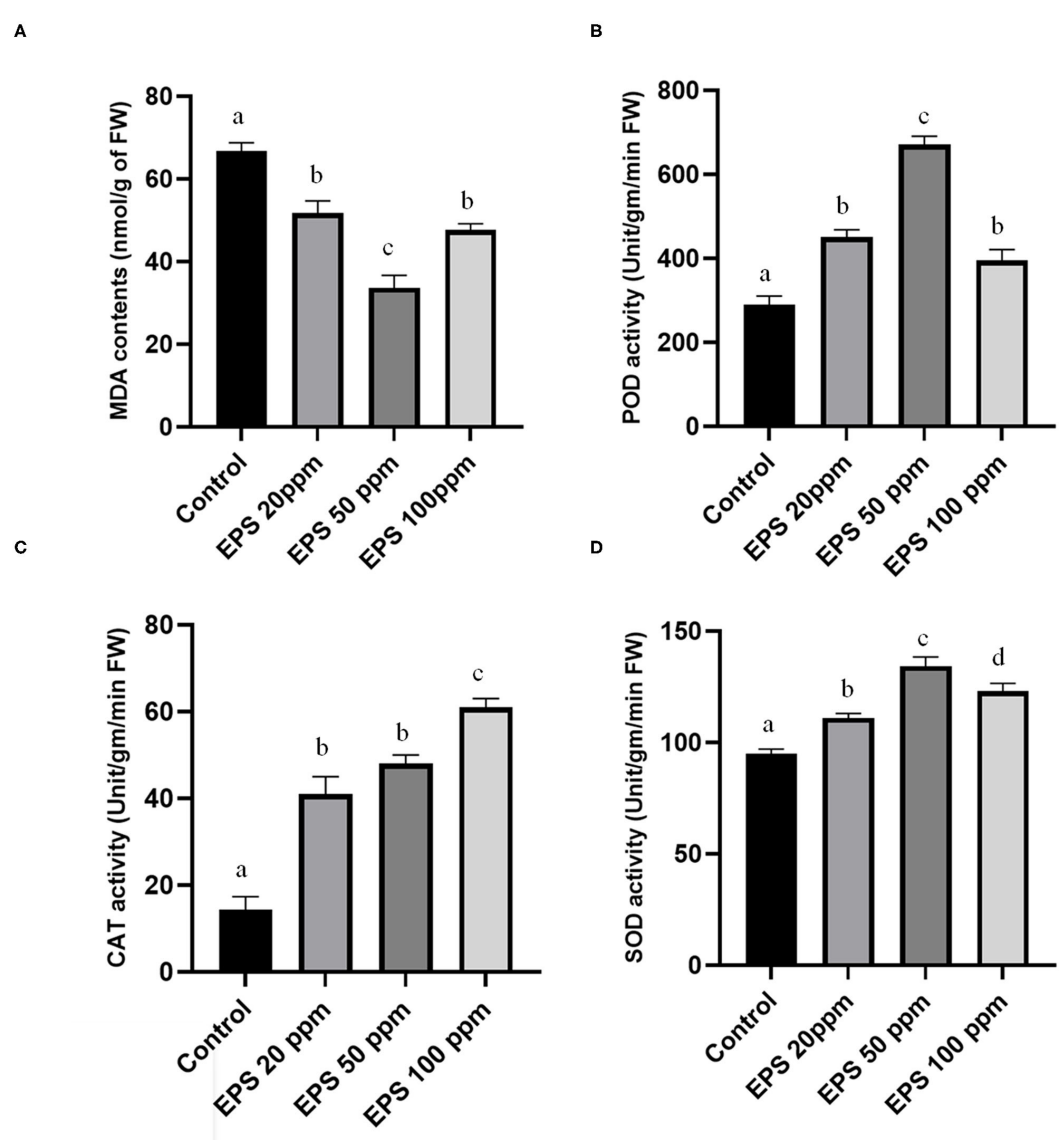
Alteration of **(A)**-malondialdehyde contents (MDA), **(B)**-peroxidase activity (POD), **(C)**-catalase (CAT) activity, **(D)**-superoxide dismutase (SOD) activity after EPS foliar spray on *Oryza sativa* ssp. *indica* MTU 7093 swarna in comparison to control. Values on the graphs are the means ± Standard error (SE) of three replicates. Tukey's multiple comparison test was performed. The different letters a, b, c, and d indicates significance differences in comparison to the control plant (At, *P* < 0.05).

Higher values of malondialdehyde contents in a plant tissue can massively affect the lipid peroxidation due to excessive reactive oxygen species generation. Colletotrichum-derived EPS reduces the malondialdehyde contents by preventing havoc lipid peroxidation, thus promoting drought stress tolerance. Malondialdehyde contents reduced from 69.3 nmol/gm of fresh weight to 51.7 (25.39% reduction), 37.1 (46.6% reduction), and 46.3 (33.1% reduction) nmol/gm fresh weight upon 20, 50, and 100 ppm EPS application, respectively, in comparison to the control set (Figure 6A).

In biological system antioxidant enzymes like SOD, catalase (CAT) and POD is necessary for proper alleviation of drought stress. EPS treated plants exhibited higher values of these three enzymes. Particularly, 1.17, 1.40, and 1.29 times higher than the control one in case of SOD activities, 3.63, 4.18, and 5.36 times higher than the control value for CAT activity, and 1.48, 2.31, and 1.35 times elevated than the control parameters for POD activity (Figures 6B–D).

## Discussions

### Exopolysaccharide From Endophytic *Colletotrichum alatae* LCS1 (Isolate of Club-Moss-Lycopodium Clavatum) Opens Up Horizon for Sustainable Agricultural Practices

Endophytes are the microbial symbionts that share valuable genes with their host plants. It is believed that endophytes have co-evolved with their host plants and have undergone vertical and/or horizontal gene sharing, and thus produces similar types of bioactive components (Strobel and Bryn, 2003). This is the reason why scientists focus on endophytes rather than plant sources for bioactive secondary metabolite production. Endophytes either directly or indirectly promotes plant growth and development in stress. Here, the selection of the host plant was made based on the criteria that the host plant (*Lycopodium clavatum*) has grown on red and dry soil of Tapobon forest which is quite uncommon and, to some extent, the endophytes have promoted their growth in such unfamiliar environment.

It is quite established that exopolysaccharides alleviate drought or salt stresses, making the rhizospheric environment more suitable for plant growth (Chen et al., 2017). So, the EPS-producing property of the isolated endophyte was selected for drought stress alleviation. Overall, 6 endophytic fungi were isolated from different parts (strobilus, stem and leaf) of the explant and *C*. *alatae* LCS1 was found to be the major EPS producer. The results derived from the present study supports the idea that endophyte-derived EPS is one of the major reasons for host plant survival in unfamiliar drought induced soil.

Rice seedlings were selected for this study as they are the most valuable and common staple food of the whole world's population especially in Asian countries like China, Japan, and India (Uga et al., 2013; Zhu, 2016). In competition with the random population explosion, the production of rice must be enhanced to meet the growing need. Rice is considered to be the most water consuming grain and draws almost 80% of the total irrigation water budget (Bouman and and Toung, 2001; Bouman et al., 2007). As a result of rapid urbanization and random industrialization, fresh water sources have become a major challenge and in Asia alone, ~42 million hectares of rice cultivation faces frequent and/or occasional drought stresses. All these situations lower the agricultural output so the focus must be on sustainable ways for reduction of water usage and maximization of utilization of arid and semi-arid areas for rice cultivation.

A huge percentage of our Earth is wasted as barren deserted land unsuitable for plantation due to drought and salinity stress. The cases of decreased precipitation and increased evaporation has enhanced the problems of drought stress (IPCC, 2018; Naumann et al., 2018; Dey et al., 2019). The recurrent cases of drought stresses (Millennium Drought-1997-1999, California drought-2011-2017) all over the major areas in the World, like Southern Australia and USA, has not only affected the two-thirds of the global populations but also disturbs the food security (FAO, 2018) and hampers the forest health (Allen et al., 2010). To find out some sustainable solution from endophytic sources, the drought stress resistance toward rice seedlings has been selected in this study. The outcome of the study emphasizes the fact that *C*. *alatae* LCS1-originated EPS promotes drought stress alleviation in case of rice seedlings. This type of study popularizes the concept of sustainable agricultural practices using endophyte as a potent tool.

### Monosaccharide Compositions of Heteropolysaccharides

Heteropolysaccharides are composed of unique types of monosaccharides that possess potent multifaceted bioactivities. In this study, *Colletotrichum alatae* LCS1 possess six different types of monosaccharides that alleviate drought stress in case of rice seedlings. In most of the cases the particular configuration of EPS determines it bioactivity. Here, glucose, arabinose, rhamnose, mannose, galactose, and fucose performs the drought resistance activity. The exact percentage of monosaccharides provide the particular bioactivity and enhances the bio-utility of *C*. *alatae* LCS1 polysaccharide. Similar types of heteropolysaccharides were reported from endophytic bacteria *Paeniacillus polymyxa* EJS-3 and fungi *Fusarium* sp. A14 with mannose, fructose, glucose and rhamnose, arabinose, glucose, and galactose with potent bioactivity, respectively (Liu et al., 2009; Pan et al., 2019). This is the first time heteropolysaccharide from *C*. *alatae* LCS1 has been reported with this type of monosaccharide compositions.

### Optimization of Necessary Fermentation Parameters

Fermentation medium needs to be optimized for the maximized production of any metabolic components. In this study, fermentation medium composition, fermentation time, medium pH, carbon source, additional nitrogen source, salts, medium volume, headspace volume, surface area, and O_2_ vectors have been optimized for the maximized production of the exopolysaccharide. Actually, the optimum production not only saves time but also enhances the efficacy of the whole fermentation system. Not only the one variable at a time system but also response surface methodology provides more time and cost-effective solution regarding this approach. Here, Exopolysaccharide derived from *Colletotrichum alatae* LCS1 was proved to be potent agent for drought stress resistance, and it could be easily exploited for large scale production and commercial use. OVAT optimization techniques promote the selection of multiple physical conditions and nutrient components that can uplift the product outcome. Here, the yield was maximized (17.38 ± 0.093 g/l EPS) up to 59% than the pre-optimized (10.25 ± 0.071 g/l EPS) condition. In similar types of studies made by Mahapatra and Banerjee (2013, 2016) in case of *Fusarium solani* SD5 and *Pestalotiopsis* sp. BC55, the fermentation conditions were optimized by OVAT system coupled with RSM at a fermentation time of 13.68 days and 3.76 days, fermentation temperature of 28 and 24°C, medium volumes of 50 and 75 ml in 250 ml Erlenmeyer flask, medium pHs of 6.46 and 6.93, additional carbon source of glucose (9.8 and 7.66 g/lit), additional nitrogen source of yeast extract (0.69 g/lit) and urea (0.29 g/it), additional salt using KCl (0.05 g/lit), KH_2_PO_4_ (0.05 g/lit), and CaCl_2_ (0.05 g/lit), respectively, in order to obtain the increased production of exopolysaccharide (from 0.96 ± 0.021 to 2.276 ± 0.032 g/lit and from 1.32 ± 0.045 to 4.320 ± 0.022 g/lit).

Another factor other than these vital parameters is the amount of dissolved oxygen. Here in this study, it has been revealed that fixing of proper O_2_ vector and addition at proper time enhances the EPS production up to 10% (15.69–17.38 g/lit). In industrial production lines, the larger the quantity, the higher the commercial acceptability, and fungi being an aerobic organism needs high values of O_2_ for optimized action (De Swaaf et al., 2003; Duan et al., 2008). The use of propellers or stirrers in fermenters or bioreactors includes a list of problems like higher involvement of energy, formation of unwanted foams, disturbance in the microbial growth, breakage of the exopolymers, and hampering of the culture viscosity but can promote EPS production to some extent (Xu et al., 2007; Liu et al., 2017a,b). So, in order to avoid this problem, liquid alkanes are added as O_2_ vectors that can enhance O_2_ solubility (15-20 times higher), promptly make the O_2_ mass transfer from the organic phase to aqueous phase – accelerating the O_2_ availability (Wei et al., 2020). In this EPS production system, the increasing concentration of liquid alkane (n-hexane-4%) improved the O_2_ absorption capacity. But increase of n-hexane concentration over a limit cause a sharp fall of EPS production probably due to higher viscosity of the fermentation medium affecting O_2_ transfer (Sun et al., 2020). N-hexane can interact with fungal membrane permeability and can hamper the EPS production. The proper time period for the addition is also necessary as the EPS is mainly produced during logarithmic phase and addition at the initiation of fermentation procedure is the best effective method (Giavasis et al., 2006). Similar types of result are found in case of lycopene (43.7% higher), β-carotene (48% higher), and phosphoglyceric acid (25% higher) optimization by addition of 10 g/lit of n-hexane and 3 g/lit of n-heptane respectively (Xu et al., 2007).

### Role of Fungal EPS in Drought Stress Alleviation

Agricultural production of valuable crops is severely affected by environmental stresses like drought, salinity, and water logging. Among them, drought is the most fatal among them. So, fruitful strategies must be adopted to check this economic loss and some way out must be pronounced for uninterrupted agricultural production (Zhu, 2016). Microorganisms or microorganism-derived products are popularly used as a tool for sustainable agricultural practices (Santra and Banerjee, 2020a). Especially that the fungal extracellular polymers are potent agent for drought stress alleviation due to their unique structural combination, dense polymeric appearance, hydrophilic nature, and maximized bio-film-forming ability (Muley et al., 2019). Polysaccharides like β-D-glucan and chitosan promote growth of potato, soybean, and barley (Luan and Uyen, 2014; Gandra et al., 2016; Muley et al., 2019).

The present study revealed that EPS-sprayed seedlings exhibit elevated proline and sugar contents and a medium dose of 50 ppm application is the optimum for these two functions. Thus, *Colletotrichum alatae* LCS1 originated heteropolysaccharide alleviates drought stress in case of rice seedlings. Soluble sugars and prolines are the typical osmolytes of plant cells that can improve osmotic imbalances providing relieve from drought stress (Hare et al., 1998). Due to higher degree of rigidity and hydrophilicity, osmolytes contribute to the structural and functional integrity of the cellular or subcellular compartments by maximizing the inflow of solvent (water) and restricting the loss of water from cell. Thus, it maintains a constant turgor pressure leading to cell expansion (Kaur and Asthir, 2015).

These osmolytes (EPS) promote removal of reactive oxygen species and free radicals, enhance the antioxidant enzyme activities (SOD, CAT, and POD), modifies the first line of antioxidant defenses, and degrades the harmful superoxide to H_2_O_2_, which finally breaks down to water and oxygen (Das and Roychoudhury, 2014). Harmful superoxide cause damage by lipid peroxidation and higher MDA content is the main culprit here, but elevation of antioxidant enzymes lowers the MDA content in cells (Miller et al., 2010) and prevent the cellular damage.

Superoxide dismutase (SOD), POD, and CAT activities were found to be elevated, and drought stress minimization was reported in case of potato plant by chitosan treatment (Muley et al., 2019). *Gandoerma lucidum* and *Lactobacillus plantarum* promoted the antioxidant enzyme activities in their respective host plants (Blainski et al., 2018; Zhang et al., 2020).

Endophytic fungi can minimize the lipid peroxidation and checks the leakage of electrolytes (Khan et al., 2012). Plants inoculated with endophytic fungi can influence the production of phytohormones and salicylic acid in order to survive in drought stress. They mediate stomatal closure, restricts excessive water loss, modifies genetic responses related to stress, increases the levels of pathogenesis related protein, increases vigor, elevates chlorophyll contents, and increases relative water contents in tissues (Jan et al., 2019; Khan et al., 2019). *Porostereumspadiceum* AGH786, an endophytic fungus, protects the soybean plants from salt stress by modifying ABA, GA, and jasmonic acid levels (Hamayun et al., 2017). Also, cucumber plants were, to some degree, protected from salt stress by the endophytic *Paecilomycesformosus*.

llyas et al. (2020) reported that exopolysaccharides from endophytic *Azospirillumbrasilense* promote growth and development of wheat plants under stress. The physical (root length, shoot length) and biochemical (IAA, ABA, GA, CK, Chl b, Chl a, and carotenoids) parameters improve assuring the stress tolerance of the treated seedlings. The antioxidant defense enzymes (SOD, CAT, and POD) also improved than the control one.

Endophytic *Phoma* sp. (from *Pinus tabuliformis*) promote drought stress resistance by elevating POD, CAT, and SOD activities (Zhou et al., 2021). *Glomus mosseae, G*. *versiforme*, and *G*. *diaphanum* (endophyte of *Poncirus trifoliata*) also promote drought stress by increasing the peroxidase activity and can also improve the soil textures (Wu et al., 2008). There are other examples of endophytic bacteria *Pantoeaalhagi* strain LTYR-11Z^T^, *Acinetobacter brumalii* strain MZ30V92, *Streptomyces geysiriensis* DE27, *Burkholderiaphytofirmans* PsJN, and *Rhizobium tropici* that reduces reactive oxygen species, reduces H_2_O_2_ values, increases ACC deaminase activity, uplifts IAA activity, accumulates sugar components, decreases proline contents, lowers malonaldehyde contents, decreases degradation of chlorophyll, upregulates drought response genes (DREB2B, DHN3, LEA-14-A), modulates DNA methylation genes (MET1B, CMT3, DRM2), and enhances POD, CAT, and SOD activities (Naveed et al., 2014; Khan et al., 2016; Chen et al., 2017; Sandhya et al., 2017; Jayakumar et al., 2020). In case of plants like tomato, potato, and cotton, the polysaccharides from *G*. *lucidum, Lactobacillus plantarum* promote systemic resistance, disease resistance, promotes stomatal closure, and reduces water evaporation (Blainski et al., 2018; Zhang et al., 2020).

## Conclusion

Sustainable development is the most appropriate nexus between progression of modern century and nature's equilibrium. Endophytes from untapped sources, places that can open up broad aspects of deep ecological approaches where biological organisms in proper guidance of advance biotechnology, can solve problems of xenobiotic toxicities, diseases, and agricultural production-related losses and can enhance our economic status. The present investigational outcomes are said to be appropriate with this giant theme of sustainable agricultural practices. Endophytes being a less explored entity in this arena is attracting world-wide attention due to their unique metabolomic profile, and exopolysaccharides are the top in that list. This study is a preliminary step to that huge movement but attains potent promises for being utilized as drought-tolerating and drought-ameliorating agent. Further, detailed genetic and biochemical studies are needed in order to evaluate the genetic responses along with epi-genetic factors. A multifaceted coupling with biology-statistics-biotechnology with classic and applied approaches can establish this *C*. *alatae* LCS1 strain as a famous drought removal EPS producer.

## Data Availability Statement

The original contributions presented in the study are publicly available. This data can be found at: MH102383.1.

## Author Contributions

HS formulated and performed experiments and prepared the manuscript. DB supervised and help in revision of the manuscript. Both authors contributed to the article and approved the submitted version.

## Conflict of Interest

The authors declare that the research was conducted in the absence of any commercial or financial relationships that could be construed as a potential conflict of interest.

## Publisher's Note

All claims expressed in this article are solely those of the authors and do not necessarily represent those of their affiliated organizations, or those of the publisher, the editors and the reviewers. Any product that may be evaluated in this article, or claim that may be made by its manufacturer, is not guaranteed or endorsed by the publisher.
